# Synthesis of a MnO_2_/Fe_3_O_4_/diatomite nanocomposite as an efficient heterogeneous Fenton-like catalyst for methylene blue degradation

**DOI:** 10.3762/bjnano.9.185

**Published:** 2018-07-06

**Authors:** Zishun Li, Xuekun Tang, Kun Liu, Jing Huang, Yueyang Xu, Qian Peng, Minlin Ao

**Affiliations:** 1 School of Minerals Processing and Bioengineering, Central South University, Changsha 410083, China; 2 Hunan Key Laboratory of Mineral Materials and Application, Central South University, Changsha 410083, China

**Keywords:** diatomite, Fenton-like oxidation, hybrid catalyst, iron(II,III) oxide (Fe_3_O_4_), manganese(IV) oxide (MnO_2_)

## Abstract

Heterogeneous Fenton-like catalysts with the activation of peroxymonosulfate (PMS), which offer the advantages of fast reaction rate, wide functional pH range and cost efficiency, have attracted great interest in wastewater treatment. In this study, a novel magnetic MnO_2_/Fe_3_O_4_/diatomite nanocomposite is synthesized and then used as heterogeneous Fenton-like catalyst to degrade the organic pollutant methylene blue (MB) with the activation of PMS. The characterization results show that the Fe_3_O_4_ nanoparticles and nanoflower-like MnO_2_ are evenly distributed layer-by-layer on the surface of diatomite, which can be readily magnetically separated from the solution. The as-prepared catalyst, compared with other Fenton-like catalysts, shows a superb MB degradation rate of nearly 100% in 45 min in the pH range of 4 to 8 and temperature range of 25 to 55 °C. Moreover, the nanocomposite shows a good mineralization rate of about 60% in 60 min and great recyclability with a recycle efficiency of 86.78% after five runs for MB. The probable mechanism of this catalytic system is also proposed as a synergistic effect between MnO_2_ and Fe_3_O_4_.

## Introduction

Organic contaminants are widely distributed in water and soil due to the excessive emissions of industrial processes, which causes a great threat to the ecosystem as well as to human health [1–3]. Most of the organic pollutants are toxic and can not be degraded spontaneously, thus various methods focusing on the removal of organic pollutant, including adsorption, photocatalysis and advanced oxidation processes, have been extensively studied over the past decades [2,4–5].

Among these methods, advanced oxidation processes (AOP) are considered as the most promising method because of the high removal efficiency and wide application scopes [6–7]. Iron-based homogeneous and heterogeneous Fenton or Fenton-like catalysts with the activation of H_2_O_2_ can effectively generate hydroxyl radicals (•OH, the main reactive species for the degradation of organic contaminants) and show the advantages of a fast reaction rate and being cost-efficient [8]. However, the practical application is limited through issues such as the narrow acidic pH range, non-selective oxidation, H_2_O_2_ storage and sludge removal [9–10]. To overcome the limitation, the heterogeneous Fenton-like system of MnO_2_-peroxymonosulfate (MnO_2_-PMS) is proposed. As alternatives to H_2_O_2_ and hydroxyl radicals (•OH), peroxymonosulfate (PMS) and the sulfate radical (•SO_4_^−^) exhibit a wider functional pH range, convenience in storage and higher oxidation selectivity. Also, compared to iron-based catalysts, MnO_2_ shows good catalytic performance because of the Mn(III)/Mn(IV) redox loop and much less sludge formation due to the neutral functional pH value [11–12].

Although, the MnO_2_-PMS system shows good prospect in the treatment of organic pollutants, modifications focusing on the catalytic performances and the recycling of the catalyst still need to be optimized. Previous researches have proved that structure and morphology of catalysts with the same chemical composition can significantly affect the catalytic activity of the catalysts [9,13]. Nano-scaled metal catalysts tend to agglomerate and are difficult to be dispersed because of the large surface energy, thus active sites on the surface of catalysts are covered, which causes huge impact on the practical catalytic performance. In order to solve the problem, loading the catalysts on appropriate carriers to form a core–shell composite is the most effective strategy [14]. Through this strategy, catalysts with controllable particle size, better dispersion and decreased agglomeration are easy to be synthesized and are expected to significantly increase the contact between active sites and contaminants. In addition, with the support of carriers, the prepared composites possess a much higher specific surface area, which further enhances the adsorption and the consecutive degradation performance of the catalysts.

Diatomite, a natural porous mineral originating from the fossilization of diatom shells, is composed of amorphous silica. Hence, diatomite offers a high specific surface area, a mesoporous structure and superior physicochemical stabilities. Due to the above advantages, together with the abundant reserves, low price and eco-friendly nature, diatomite is a promising support material for catalyst nanoparticles in practical applications [15–17]. In addition to stability, separability is an extremely crucial factor for the recycling of catalysts. Magnetic separation is very attractive in the field of wastewater treatment as it provides a convenient and cost-effective way for catalyst collection. Fe_3_O_4_ is a very popular magnetic material being systematically researched in various aspects from drug delivery to catalyst separation [18]. As a typical heterogeneous Fenton-like catalyst in H_2_O_2_ activation systems, it is reported that when coupling with MnO_2_, the Fe_3_O_4_–MnO_2_ pair presents a synergistic effect, which can significantly enhance the catalytic performance compared to the single-component catalysts [10]. In addition, the synergistic effect of the composite is deeply influenced by the contact area between the two phases, thus the core–shell structure of catalysts can dramatically magnify the contact area and further strengthen the synergistic effect from the structural aspect.

Herein, we propose a novel nanocomposite of magnetic MnO_2_/Fe_3_O_4_/diatomite as an efficient heterogeneous Fenton-like catalyst for PMS activation. In this study, the catalytic activity of the nanocomposite is evaluated systematically by decomposing methylene blue (MB) as the target pollutant, because MB is a typical organic dye with well-known toxicity and a threat to water environments [19]. The structural characterization and recyclability of the nanocomposite are also investigated. To the best of our knowledge, there are no reports about the synthesis of diatomite-based MnO_2_/Fe_3_O_4_ nano-materials for Fenton-like reactions.

## Experimental

### Materials and reagent

Iron(III) acetylacetonate (Fe(acac)_3_), peroxymonosulfate (PMS, KHSO_5_·0.5KHSO_4_·0.5K_2_SO_4_) were purchased from the Aladdin Industrial Corporation (Shanghai, China). Potassium permanganate (KMnO_4_) was obtained from the Xilong Chemicals Reagent Company (Shantou, China). Methylene blue trihydrate (MB, the chemical structure is shown in Figure S1, Supporting Information File 1) and triethylene glycol (TREG) were obtained from Sinopharm Chemical Reagent Company (Shanghai, China). HCl and NaOH were purchased from Tianjin Guangfu Fine Chemical Institute (Tianjin, China). All chemicals used in the work were of analytical grade and used without any further purification. Raw diatomite was obtained from Linjiang City, Jilin Province, China. Deionized water was used throughout this study.

### Synthesis of catalyst

Raw diatomite was purified by acid-washing in 2 M HCl solution at 75 °C for 4 h according to [20]. The Fe_3_O_4_/diatomite composite was prepared through thermal decomposition and in situ loading [3]. Typically, 0.4 g of purified diatomite was first put into 60 mL TREG under magnetic stirring for 30 min. Then 600 mg of Fe(acac)_3_ was added as the iron precursor under further 30 min of stirring. The obtained mixture was heated to 278 °C at a rate of 3 °C·min^−1^, and kept at reflux for 30 min under vigorous stirring and under N_2_ protection. After cooling, the prepared composite was washed with ethanol and water for three times, then the resulting Fe_3_O_4_/diatomite was dried in vacuum for further application.

The MnO_2_/Fe_3_O_4_/diatomite nanocomposite was synthesized by hydrothermal synthesis starting from Fe_3_O_4_/diatomite [13]. Dried Fe_3_O_4_/diatomite was added into 70 mL water and stirred vigorously for 30 min with subsequent ultrasound dispersion for 5 min, followed by dissolving 0.35 g KMnO_4_ into the mixture. The resulting mixture was transferred into a 100 mL autoclave and kept at 160 °C for 12 h under slight stirring. Finally, the MnO_2_/Fe_3_O_4_/diatomite nanocomposite was obtained after washing with water and drying in vacuum at 60 °C.

### Characterization methods

The X-ray powder diffraction (XRD) patterns of the samples were measured with a Bruker AXS D8 advance X-ray powder diffractometer utilizing a Cu Kα source (λ = 0.15418 nm). The functional groups of materials were characterized by Fourier transform infrared spectroscopy (FTIR) using a Nicolet Nexus 670 spectrometer. The morphology of samples was observed with a TESCAN MIRA3 LMU scanning electron microscope (SEM) and a JEOL JEM-1200EX transmission electron microscope (TEM). The X-ray photoelectron spectroscopy (XPS) measurements were carried out in an ultra-high vacuum VG ESCALAB250Xi electron spectrometer. The magnetic properties of the samples were performed by a Lakeshore 7404 vibrating sample magnetometer (VSM). The BET measurements of the samples were collected by a Micromeritics ASAP 2020 surface area and porosimetry system with N_2_ at 77 K. The total organic carbon (TOC) of the catalytic regeneration system was measured with a Shimadzu TOC-L analyzer.

### Evaluation of catalytic performance

The catalytic MB removal experiments were carried out in a 250 mL flask at constant temperature in a water bath. Typically, the catalyst was mixed with 200 mL of MB solution (10 mg/L) and stirred for 30 min to achieve adsorption equilibrium. Then, PMS (0.06 g) was added to the solution to activate the catalytic reaction. The initial pH values of the solution were adjusted by dilute NaOH and HCl solutions (1 mol/L). The water samples were taken with a syringe with filter membrane (0.45 μm) at predetermined times and analyzed by a UV-2600 spectrophotometer at the absorption wavelength of 664 nm. The effects of reaction temperature, pH value and PMS dosage on the catalytic reaction were also researched under same experimental conditions. Each point in all plots is the average value from three replicate experiments.

### Recyclability test

The used catalysts after MB removal were magnetically collected for the next cycle of catalytic reaction, the experimental procedures and parameters were exactly same as those of the above removal experiments. The recycle efficiency (*E*_R_, %) was evaluated by comparing the removal rate performances of fresh and used catalyst.

## Results and Discussion

### Structural and morphological characterizations

**Figure 1:**
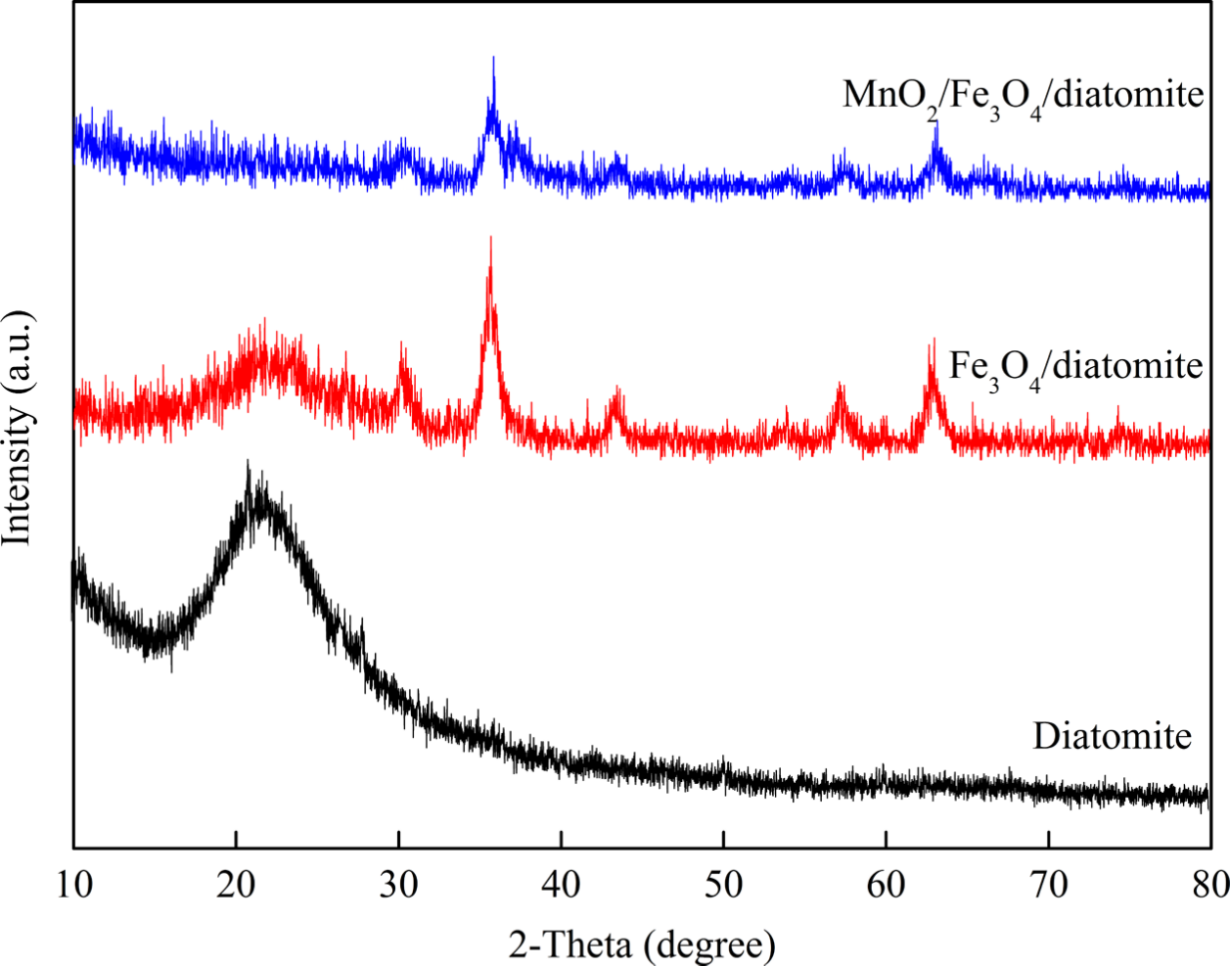
XRD patterns of the purified diatomite and the prepared diatomite-supported composites.

Figure 1 shows the XRD patterns of purified diatomite, Fe_3_O_4_/diatomite and MnO_2_/Fe_3_O_4_/diatomite. For diatomite, a broad peak at around 22.5° is observed due to the amorphous structure of SiO_2_. No other obvious diffraction peaks are detected, suggesting that after the washing, the raw diatomite is highly pure. Six peaks at 29.9°, 35.3°, 43.2°, 53.3°, 57.00° and 62.4° are observed in the pattern of Fe_3_O_4_/diatomite. These peaks match well with the (220), (311), (400), (422), (511) and (440) plane spacings of cubic magnetite (JCPDS file No. 19-0629), suggesting the Fe_3_O_4_ nanoparticles are successfully coating the surface of diatomite [21]. In addition, the characteristic peaks of magnetite have a large FWHM, revealing the small size of the loaded Fe_3_O_4_ particles. The average crystal size of the Fe_3_O_4_ nanoparticles estimated with the Scherrer formula is about 10 nm. After the hydrothermal treatment with KMnO_4_, no new characteristic peaks are detected in the pattern of the as-prepared MnO_2_/Fe_3_O_4_/diatomite. However, the original characteristic peaks of magnetite are weakened. This indicates that the nano-MnO_2_ covering on the Fe_3_O_4_/diatomite is of low crystallinity or amorphous.

**Figure 2:**
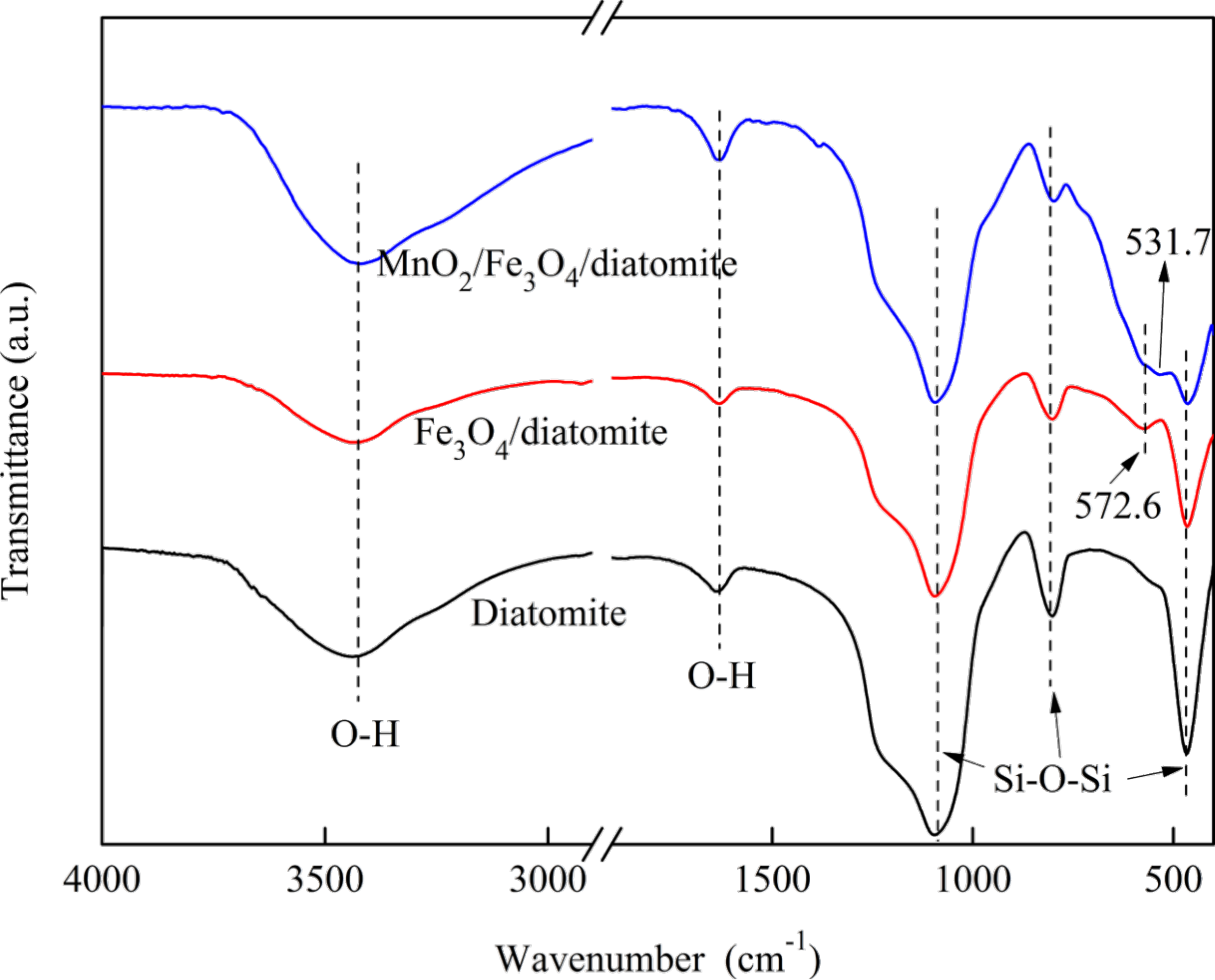
FTIR spectra of the purified diatomite and prepared diatomite-supported composites.

Figure 2 shows the FTIR spectra of purified diatomite, Fe_3_O_4_/diatomite and MnO_2_/Fe_3_O_4_/diatomite. The broad peaks at 3400 and 1630 cm^−1^ that are observed in all three samples are typically related to stretching vibrations of hydroxyl (–OH) groups on the surface of the samples [22]. The peaks at 1094, 804 and 467 cm^−1^ are attributed to asymmetric stretching, symmetric stretching and bending modes of Si–O–Si bonds in diatomite [23–25]. For Fe_3_O_4_/diatomite and MnO_2_/Fe_3_O_4_/diatomite, the peak at 572.6 cm^−1^ is assigned to stretching vibrations of Fe–O–Fe, indicating the loading of Fe_3_O_4_ nanoparticles on the composite [26]. The peak at 531.7 cm^−1^ can be ascribed to the Mn–O vibrations of MnO_2_ [27].

**Figure 3:**
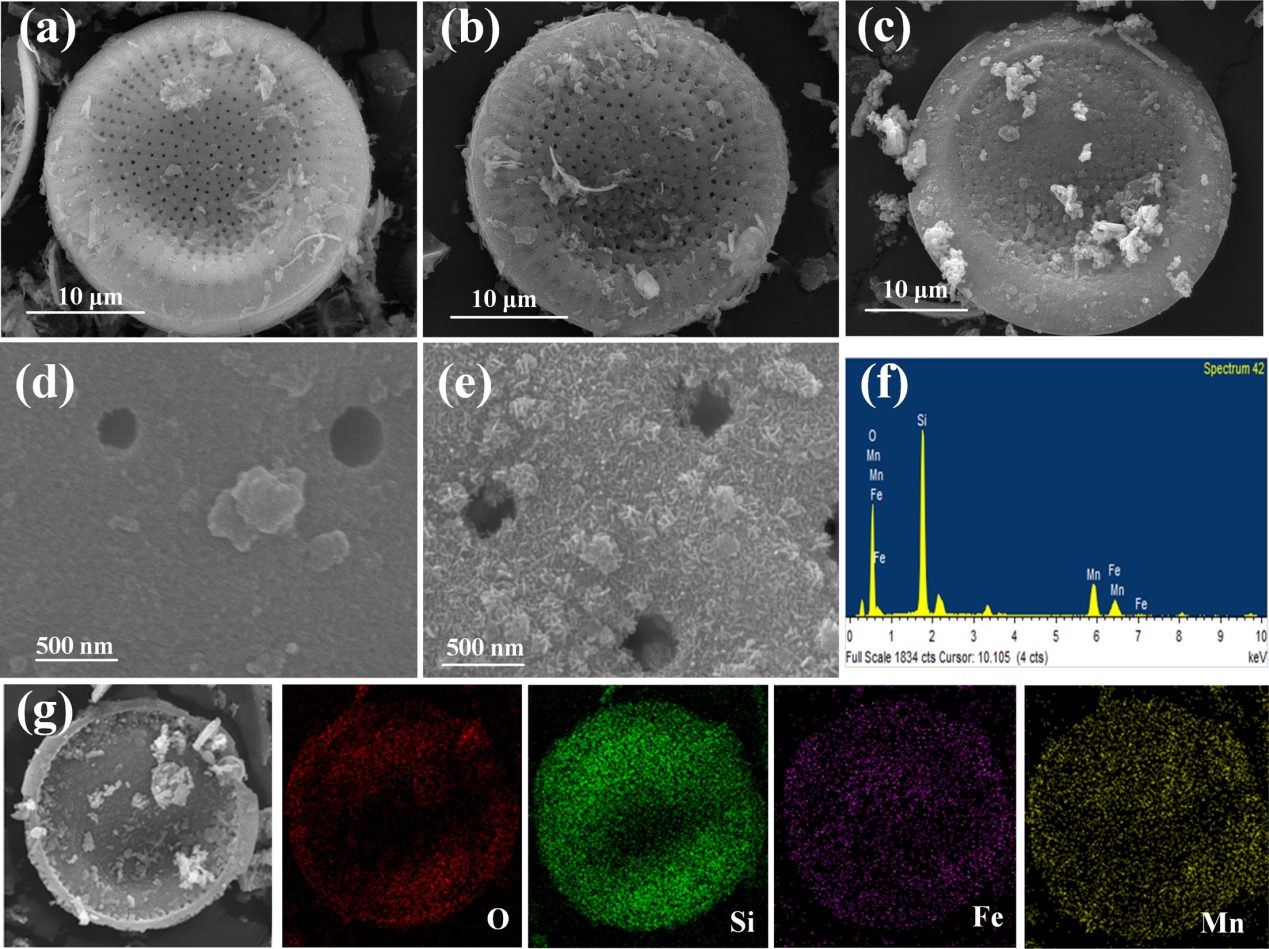
The SEM images of diatomite (a), Fe_3_O_4_/diatomite (b, d), MnO_2_/Fe_3_O_4_/diatomite (c and e), EDX spectrum (f) and elemental mapping images (g) of MnO_2_/Fe_3_O_4_/diatomite.

Figure 3 shows the SEM images of the samples at different synthesis stages. As it can be seen, the purified diatomite shows a typical plate-like morphology with a diameter of about 25 μm, and abundant pores with sizes of about 0.2 μm are evenly distributed on the surface of the diatomite, which is beneficial for the controllable assembly of composites and their application in wastewater treatments. Then, the as-prepared Fe_3_O_4_/diatomite shows a slightly rough surface and a part of the pores are blocked by the attachments, indicating the evenly loading of the tiny Fe_3_O_4_ nanoparticles. For the MnO_2_/Fe_3_O_4_/diatomite, a rather rough surface is observed after the hydrothermal treatment with KMnO_4_, and almost all the pores are blocked by a layer of MnO_2_. As shown in the magnified image of MnO_2_/Fe_3_O_4_/diatomite (Figure 3e), the prepared nanosized MnO_2_ shows a flower-like or urchin-like structure on the surface of diatomite. Meanwhile, the plate-like morphology of diatomite is well preserved after the two-step loading process, revealing the negligible influence of the hydrothermal process on the structure of diatomite. In addition, Figure 3f and Figure 3g present SEM images of the MnO_2_/Fe_3_O_4_/diatomite composite and the corresponding energy-dispersive X-ray (EDX) spectrum and elemental mappings for O, Si, Fe and Mn elements, respectively. It can be found that all the elements are evenly distributed on the diatomite, and the calculated atomic fractions of Fe and Mn are 3.63% and 10.96%, respectively. All these results confirm the successful loading of of iron oxide and manganese oxide in the two-step procedure.

**Figure 4:**
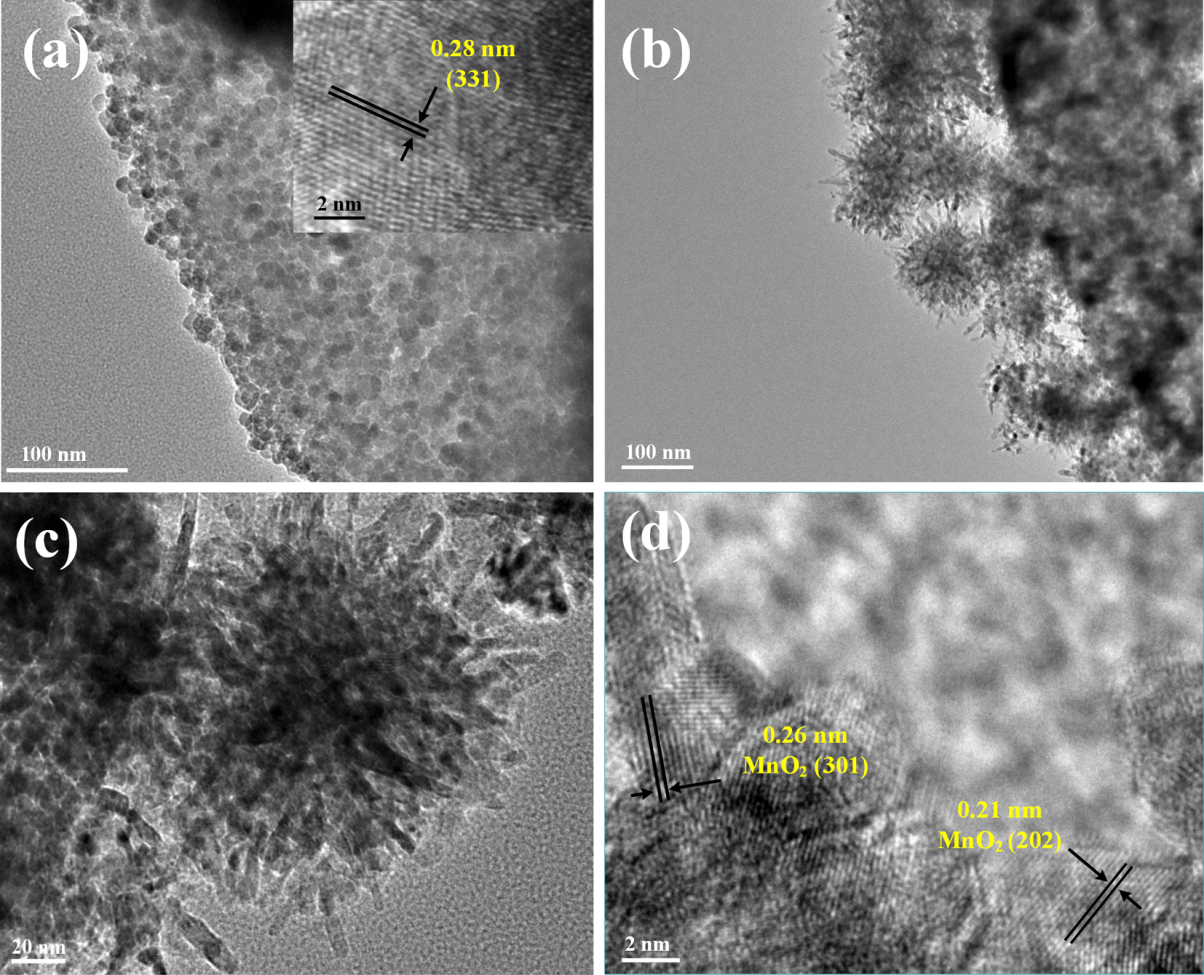
TEM images of (a) Fe_3_O_4_/diatomite, (b, c) MnO_2_/Fe_3_O_4_/diatomite and (d) HRTEM image of MnO_2_/Fe_3_O_4_/diatomite.

To further characterize the morphologies and structures of Fe_3_O_4_/diatomite and MnO_2_/Fe_3_O_4_/diatomite, transmission electron microscopy (TEM) and high-resolution transmission electron microscope (HRTEM) analyses were also performed. As shown in Figure 4a, the Fe_3_O_4_/diatomite exhibits a morphology similar to that seen in the SEM images, a layer of nanoparticles with diameter of about 10 nm are highly dispersed on the surface of diatomite, which demonstrates a porous polycrystalline structure composed of many interconnected nanoparticles [28]. The marked lattice fringe spacing of 0.28 nm in the HRTEM images (inset) is corresponding to the (331) planes of cubic magnetite [29]. Figure 4b shows the TEM images of MnO_2_/Fe_3_O_4_/diatomite, the nanoparticles on the surface are fully covered by a layer of rough 3D structured material. As seen in the magnified image (Figure 4c), a flower-like or urchin-like structure of the outer MnO_2_ shell can be easily observed. The crystal structure of the outer shell is analyzed by using HRTEM, as shown in Figure 4d. As a whole, the chaotic and unclear lattice fringes in the image illustrate the poor crystallinity of MnO_2_, which is consistent with the XRD result. In some small parts of the area, however, typical spacings of 0.21 and 0.26 nm are measured, which corresponds to the (202) and (301) planes of MnO_2_ [30]. The morphology of pure MnO_2_ particles was also researched (Figure S2, Supporting Information File 1), the SEM and TEM images show that the pure MnO_2_ agglomerates into large micrometer-sized particles, in contrast to the monodisperse nano-MnO_2_ in the MnO_2_/Fe_3_O_4_/diatomite composite. The TEM analysis confirms the successful synthesis of the core–shell structured MnO_2_/Fe_3_O_4_/diatomite composite and the dispersion improvement of nano-MnO_2_ due to the introduction of diatomite.

**Figure 5:**
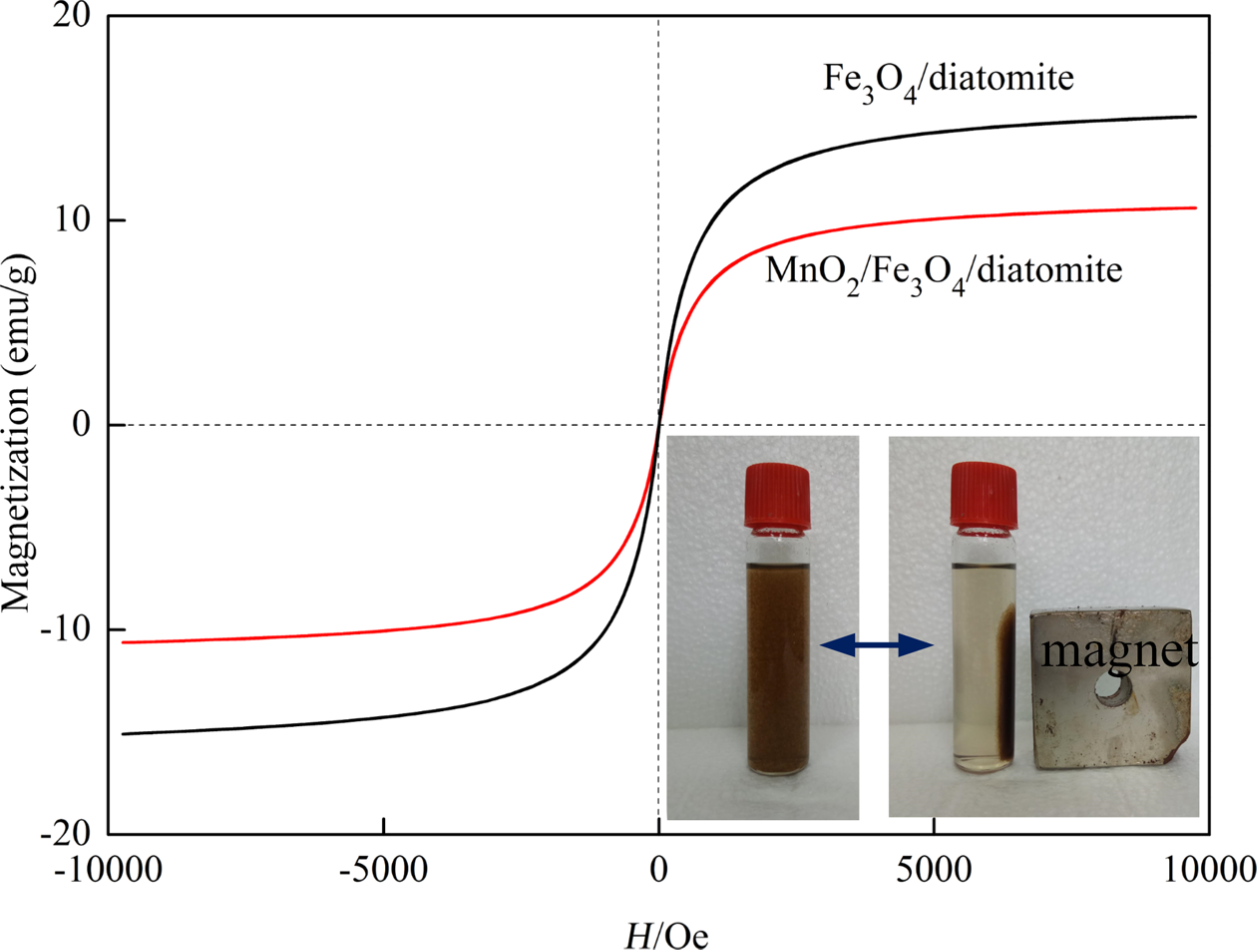
Magnetic hysteresis loops of Fe_3_O_4_/diatomite and MnO_2_/Fe_3_O_4_/diatomite. The inset image illustrates the magnetic separation of the dispersed MnO_2_/Fe_3_O_4_/diatomite.

Figure 5 plots the magnetization curves of the as-synthesized Fe_3_O_4_/diatomite and MnO_2_/Fe_3_O_4_/diatomite at a maximum field of 10 kOe at room temperature. No obvious remanence or coercivity is observed, which indicates that both of the samples show superparamagnetic behavior at room temperature [31]. The maximum saturation magnetizations of Fe_3_O_4_/diatomite and MnO_2_/Fe_3_O_4_/diatomite were measured to be 16.57 and 10.61 emu/g, respectively, which make the composites very easy to be separated by an external magnetic field (inset). The decrease of the maximum saturation magnetizations after the treatment with KMnO_4_ is largely ascribed to the outer MnO_2_ shell, the saturation magnetization of which is much lower than the Fe_3_O_4_ nanoparticles.

**Figure 6:**
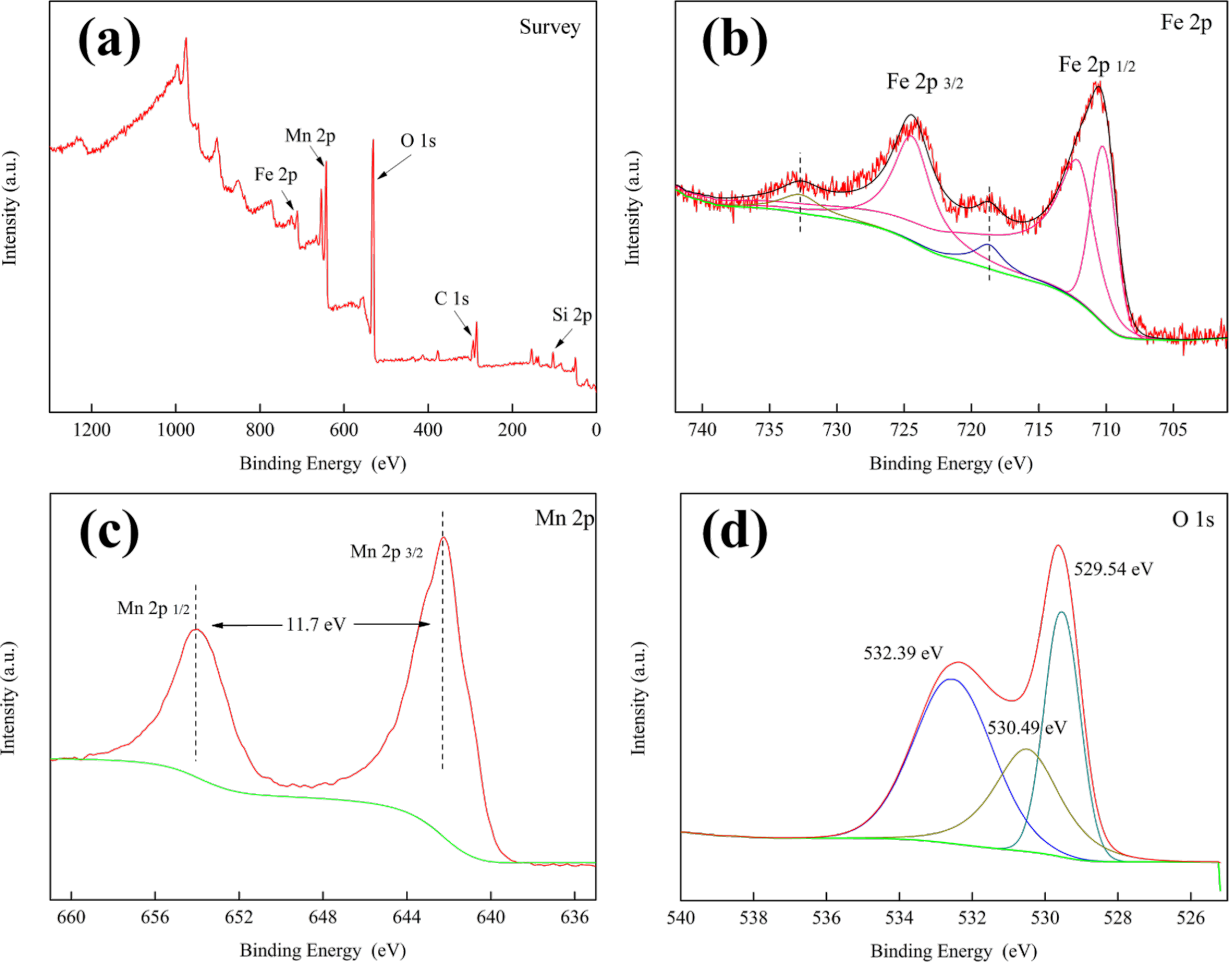
XPS spectra of as-synthesized MnO_2_/Fe_3_O_4_/diatomite: (a) full survey spectrum, high-resolution scans of (b) Fe 2p, (c) Mn 2p and (d) O 1s.

XPS measurements were carried out to determine the surface chemical compositions and the valence states of MnO_2_/Fe_3_O_4_/diatomite, and the spectra are shown in Figure 6. The Si 2p, O 1s, Fe 2p and Mn 2p peaks detected in the survey spectrum (Figure 6a) indicate the existence of Si, Fe, Mn, and O. The high-resolution scans for Fe 2p, Mn 2p and O 1s are also represented in Figure 6b–d. In Figure 6b, two peaks with binding energies of 710.8 and 724.1 eV are assigned to Fe 2p_3/2_ and Fe 2p_1/2_ peaks, which are mainly due to the FeO and Fe_2_O_3_; moreover, satellite peaks at 719.32 eV and 732.8 eV can be observed. These are the typical characteristics of the Fe_3_O_4_ structure [32]. The Mn 2p region (Figure 6c) exhibits two individual peaks at 653.9 and 642.2 eV, attributed to the Mn 2p_1/2_ and Mn 2p_3/2_ binding energies, respectively. As a result, the spin energy separation of Mn 2p peaks can be calculated as 11.7 eV, which is well in agreement with reports for MnO_2_ [33]. In Figure 6d, the O 1s scan can be fitted into three symmetric peaks located at 532.39, 530.49 and 529.54 eV. Among them, the peaks at 532.9 and 530.49 eV are assigned to the oxygen in SiO_2_ and Fe_3_O_4_, and the peak located at 529.54 eV is ascribed to lattice oxygen (Mn–O–Mn bond) in MnO_2_ [34]. Therefore, the surface chemical composition results obtained from the XPS analysis ensure the formation of Fe_3_O_4_ and MnO_2_, which further confirms the observation from the previous structural and morphological characterization.

**Figure 7:**
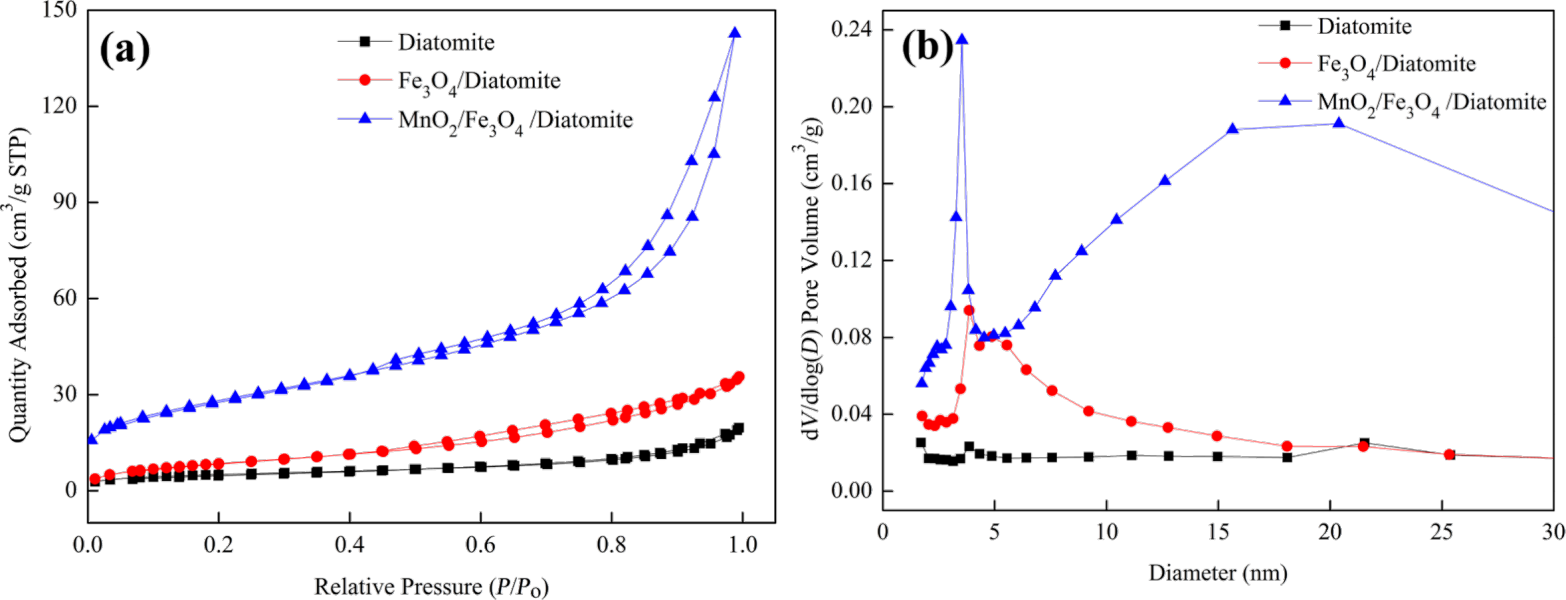
(a) N_2_ adsorption–desorption isotherms and (b) the corresponding pore-size distribution curves of diatomite, Fe_3_O_4_/diatomite and MnO_2_/Fe_3_O_4_/diatomite.

Figure 7 shows the N_2_ adsorption–desorption isotherms and corresponding pore-size distributions of the samples. All three samples show a type-IV isotherm with type-H3 hysteresis loops (at about 0.50–0.99), which demonstrates a mesoporous structure of diatomite and as-prepared composites [35]. The detailed BET values calculated by the Barret–Joyner–Halenda (BJH) method are listed in Table 1. As it can be seen, both the specific surface area and pore volume values of the samples follow the order of diatomite < Fe_3_O_4_/diatomite < MnO_2_/Fe_3_O_4_/diatomite, which implies that the loaded Fe_3_O_4_ and amorphous MnO_2_ nanoparticles exhibit porous structures just as observed in the TEM images. The MnO_2_/Fe_3_O_4_/diatomite with high surface area and sufficient mesoporous structure will provide adequate active sites in the consecutive adsorption and heterogeneous Fenton-like reaction for dye removal.

**Table 1:** BET values calculated by the BJH methods of the three samples.

sample	specific surface area (m^2^·g^−1^)	pore volume (cm^3^·g^−1^)	average pore diameter (nm)

diatomite	20.99	0.03	6.95
Fe_3_O_4_/diatomite	47.99	0.06	6.92
MnO_2_/Fe_3_O_4_/diatomite	103.55	0.22	8.81

### Catalytic activity

**Figure 8:**
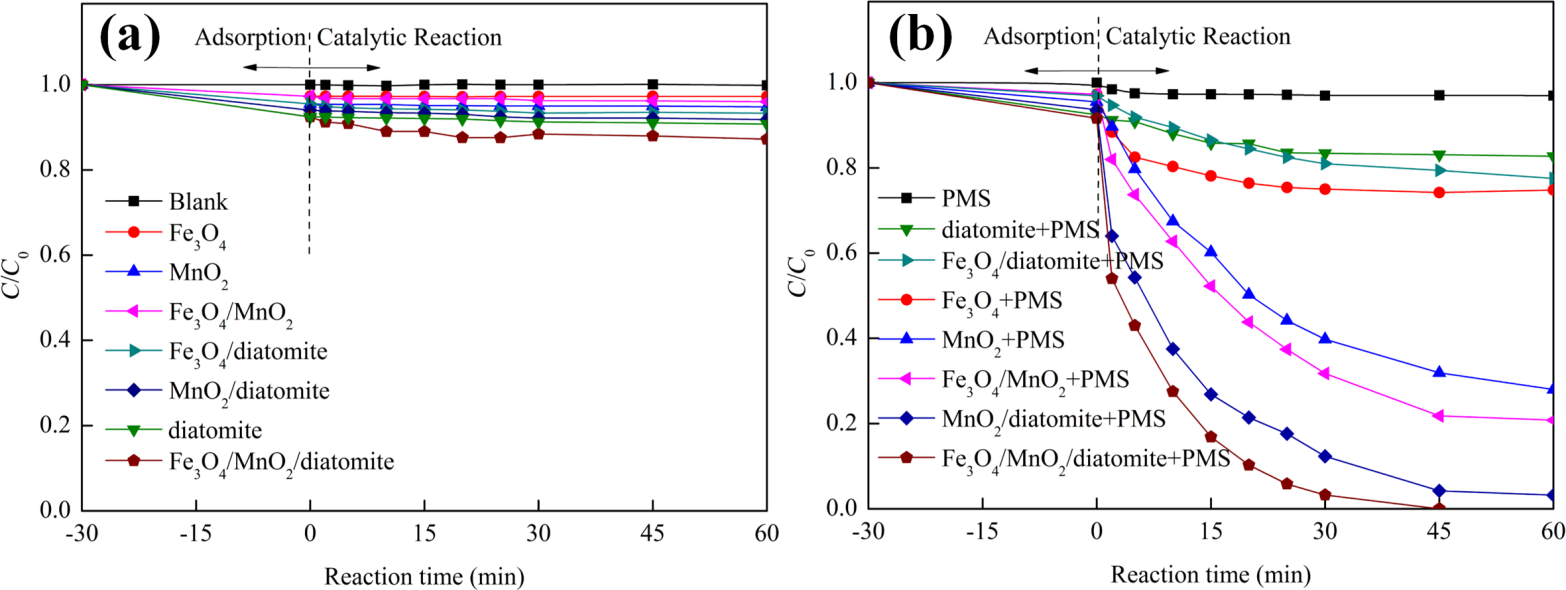
MB removal of different catalytic systems (a) without and (b) with (b) PMS. (Reaction conditions: catalyst dosage = 0.10 g/L, PMS dosage = 0.30 g/L, initial MB concentration = 10 mg/L, *T* = 25 °C, initial pH 6).

To demonstrate the catalytic activity of the as-synthesized catalysts toward PMS activation, MB was selected as the target contaminant for degradation. Figure 8 shows the comparison of adsorption and degradation removal of various materials under the same experimental conditions, the blank solution and mono-catalysts including PMS, diatomite, Fe_3_O_4_, Fe_3_O_4_/diatomite, MnO_2_, MnO_2_/diatomite, Fe_3_O_4_/MnO_2_ and MnO_2_/Fe_3_O_4_/diatomite are also evaluated. As shown in Figure 8a, all catalyst systems display negligible adsorption removal in the initial adsorption process, and less than 10% MB was removed using the different systems. Among all materials, the MnO_2_/Fe_3_O_4_/diatomite exhibits the best adsorption efficiency compared to others, which may be ascribed to its higher surface area and pore structure as shown in the BET results. In the following so-called catalysis reaction without PMS, low removal efficiencies are observed in different mono-catalyst systems over the course of 60 min, revealing that the MB can not be degraded by these Fenton-like catalysts without the activation of PMS.

For systems with Fenton-like catalysts and added PMS (Figure 8b), the MnO_2_/Fe_3_O_4_/diatomite exhibits a superb degradation rate of nearly 100% in 45 min. Compared to other catalysis systems (including Fe_3_O_4_–PMS, Fe_3_O_4_/diatomite–PMS, MnO_2_–PMS, MnO_2_/diatomite–PMS, Fe_3_O_4_/MnO_2_–PMS), the MnO_2_/Fe_3_O_4_/diatomite–PMS system exhibits an outstanding catalytic performance in MB removal, which is mainly ascribed to more exposed active sites of the monodispersed catalysts supported by diatomite. In addition, the better adsorption behavior of MnO_2_/Fe_3_O_4_/diatomite may also contribute to the subsequent catalytic reaction. A limited catalytic efficiency is observed in Fe_3_O_4_/diatomite–PMS systems, indicating that the loading of nano-MnO_2_ plays a key role in the improvement of the catalytic performance. Additionally, the MnO_2_/Fe_3_O_4_/diatomite–PMS shows a better MB removal performance than MnO_2_/diatomite–PMS, and the MnO_2_/Fe_3_O_4_–PMS performs better than MnO_2_–PMS, which shows that the Fe_3_O_4_–MnO_2_ pair is a synergistic catalyst combination for Fenton-like catalysis. The core–shell structure of the MnO_2_/Fe_3_O_4_/diatomite composite highly increases the contact area between Fe_3_O_4_ nanoparticles and nano-MnO_2_ shell, which explains well the huge activity improvement after the coating with MnO_2_.

**Figure 9:**
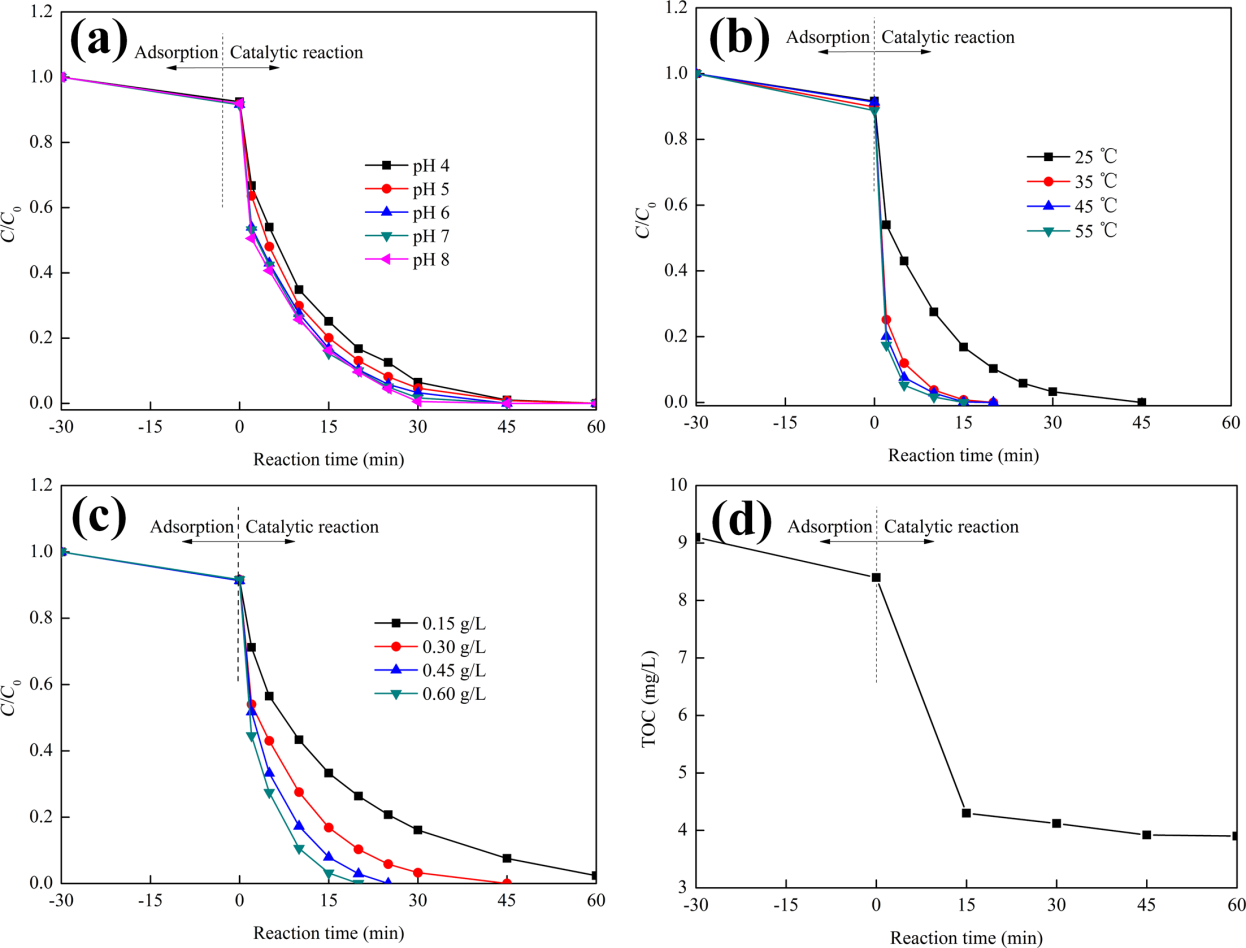
MB removal using MnO_2_/Fe_3_O_4_/diatomite at (a) different pH values, (b) different temperatures and (c) different PMS concentrations; (d) the TOC as a function of reaction time (reaction conditions: catalyst dosage = 0.10 g/L, MB concentration = 10 mg/L; default conditions: PMS dosage = 0.30 g/L, *T* = 25 °C, initial pH 6).

The functional pH range is of vital importance for the practical application of various catalytic systems, because it affects surface charge, functional groups and relative adsorption behavior of the dispersed catalysts. The application of homogeneous and heterogeneous iron-based Fenton or Fenton-like catalysts is limited to some extent by the narrow acidic pH range of the system itself. MnO_2_ works in a much wider functional pH range compared to other catalysts [36]. In this study, the influence of the pH value on the MB degradation performance of MnO_2_/Fe_3_O_4_/diatomite was investigated and the results are shown in Figure 9a. The degradation rates of MB under different pH conditions are largely the same, which demonstrates that MnO_2_/Fe_3_O_4_/diatomite is a pH-independent catalyst possessing a wide functional pH range for the activation of PMS. This phenomenon can be explained by the stability of PMS under different pH conditions in aqueous solution [37].

The influence of the reaction temperature on the degradation efficiency of the MnO_2_/Fe_3_O_4_/diatomite–PMS system is shown in Figure 9b. MB is totally degraded after 45, 17, 20 and 15 min at temperatures of 25, 35, 45 and 55 °C, respectively. Generally, a higher temperature will accelerate the removal rate of MB, indicating an endothermic nature of this heterogeneous Fenton-like process. The enitial concentration of PMS plays an essential role in the degradation process. Figure 9c shows the MB degradation results as a function of the initial PMS concentration in the MnO_2_/Fe_3_O_4_/diatomite–PMS system*.* The time required to completely remove MB decreases from 65 to 20 min along the concentration of PMS increasing from 0.15 to 0.60 g/L, which is probably due to the higher PMS concentration promoting the generation of active radicals (•SO_4_^−^). Figure 9d shows the TOC reduction as a function of the reaction time. Less than 10% of TOC is removed in the initial adsorption process. Then, the quantity of TOC decreases significantly in the subsequent 15 min of heterogeneous Fenton-like reaction and reaches the degradation equilibrium within about 45 min. Finally, in the MB degradation experiments, a TOC removal of 57.2% is obtained after 60 min, indicating the high catalytic efficiency of MnO_2_/Fe_3_O_4_/diatomite with the activation of PMS.

### Recyclability of the catalyst

**Figure 10:**
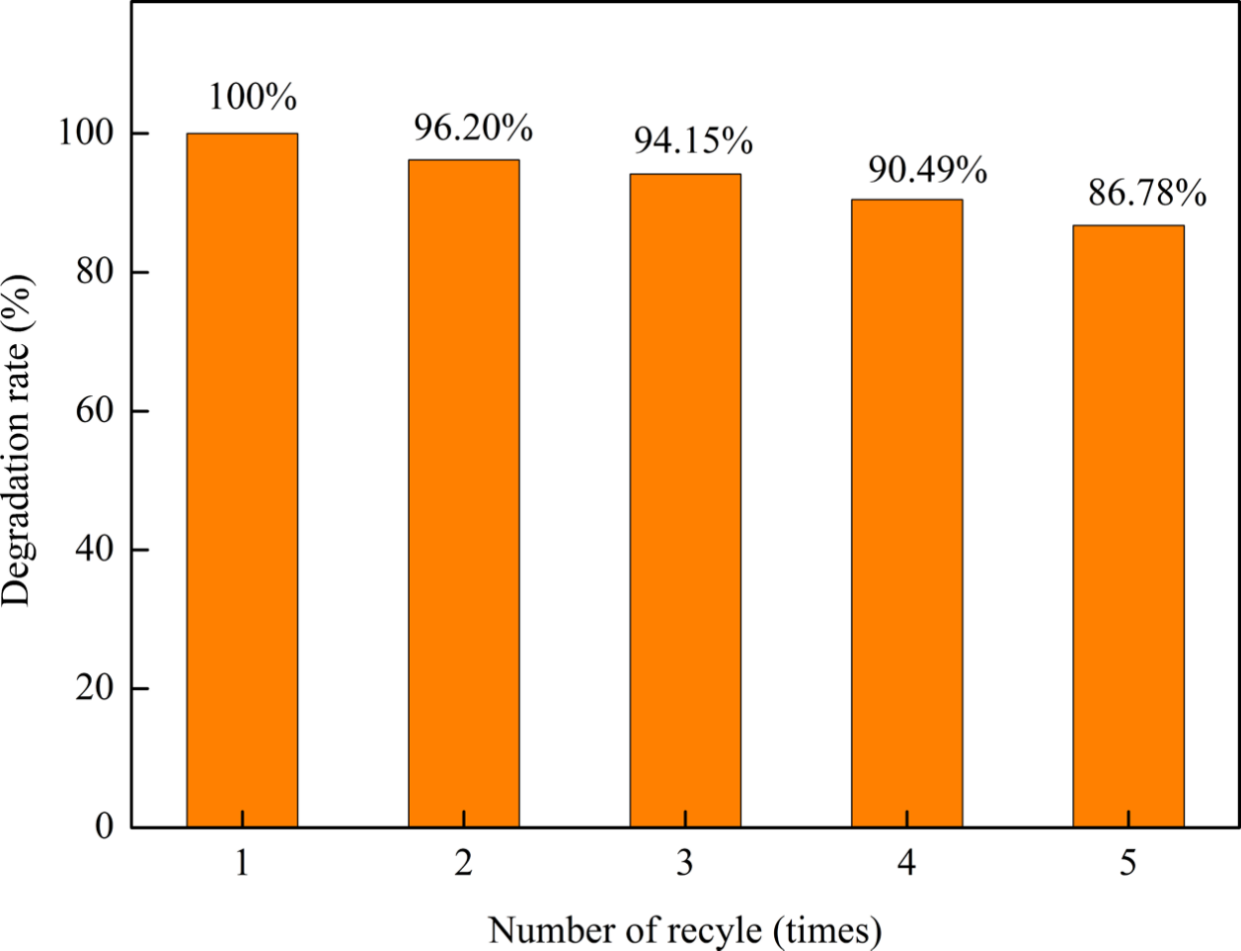
Recyclability of the MnO_2_/Fe_3_O_4_/diatomite catalyst for the degradation of MB.

The recyclability of the MnO_2_/Fe_3_O_4_/diatomite catalyst was examined by cyclically reusing the material under the same experimental conditions and the results are shown in Figure 10. The catalyst maintains a high recyclability of 86.78% after five cycles. The slight reduction of recyclability is mainly ascribed to the mass loss in long-term tests [38–39]. The outstanding recyclability of the catalysts can be explained by two aspects: (i) The core–shell structure of MnO_2_/Fe_3_O_4_/diatomite ensures the physical stability under the mechanical stirring. (ii) The catalyst in this heterogeneous Fenton-like system is easier to stabilize at the moderate catalytic conditions (without heating or acid soaking).

### Probable reaction mechanism

**Figure 11:**
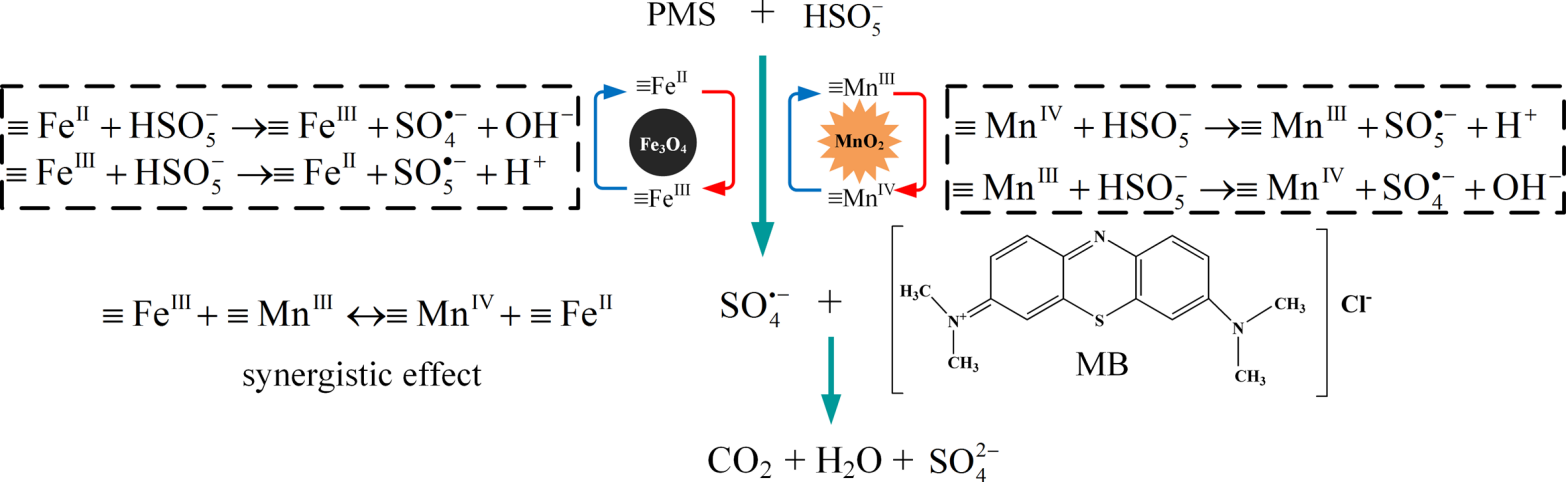
Schematic diagram of the synergistic effect between MnO_2_ and Fe_3_O_4_.

Based on the core–shell structure of the MnO_2_/Fe_3_O_4_/diatomite, a plausible catalytic mechanism is proposed and schematically illustrated in Figure 11, which considers the different shells leading to different catalytic reactions during the MB degradation. The combined catalytic system is divided into two sections, namely, the outer MnO_2_–PMS system and the inner Fe_3_O_4_–MnO_2_ pair system. The outer layer is a homogeneous shell of well-dispersed MnO_2_ nanoparticles. Based on its many active sites, active radicals are generated from PMS for the degradation of MB. The reactions of the Mn(IV)/Mn(III) redox loop are given in Equations 1–4 (≡Mn(IV) and ≡Mn(III) represent Mn(IV) and Mn(III) at the surface of MnO_2_):

[1]
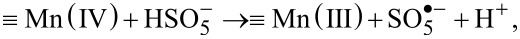


[2]
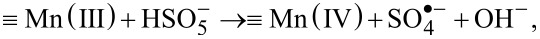


[3]
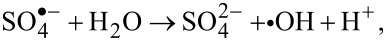


[4]
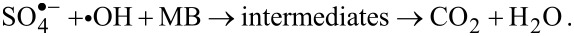


The inner layer is composed of MnO_2_ and Fe_3_O_4_. Both MnO_2_ and Fe_3_O_4_ can react with PMS to generate active radicals. Typically, the catalytic reactions are as given in Equations 5–8 (≡Fe(II) and ≡Fe(III) represent Fe^2+^ and Fe^3+^ at the surface of Fe_3_O_4_):

[5]
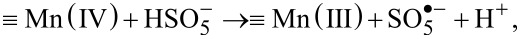


[6]
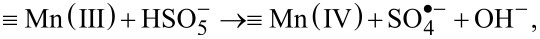


[7]
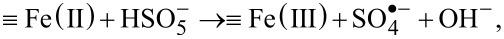


[8]
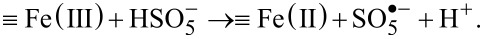


The synergistic effect between MnO_2_ and Fe_3_O_4_ accelerates the redox reactions of Fe(III)/Fe(II) and Mn(IV)/Mn(III), resulting in an accelerated •SO_4_^−^ generation rate. The standard redox potential of Mn(IV)/Mn(III) is 0.15 V [40], while that of Fe(III)/Fe(II) is 0.77 V [10]. Therefore, the transfer of electrons from ≡Mn(III) to ≡Fe(III) is thermodynamically favored. The corresponding reaction is

[9]
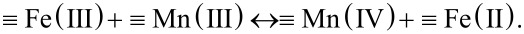


## Conclusion

A novel magnetic MnO_2_/Fe_3_O_4_/diatomite core–shell composite was synthesized. The MnO_2_/Fe_3_O_4_/diatomite showed greatly enhanced Fenton-like catalytic activity with the activation of PMS in MB degradation tests. More importantly, the MnO_2_/Fe_3_O_4_/diatomite–PMS catalytic system was almost pH-independent over a wide range. Benefiting from the core–shell structure and the neutral experimental conditions, the systems showed an excellent recyclability of 86.78% after five use cycles. A plausible mechanism of the catalytic reaction for the activation of PMS was proposed takign into account the high specific area of the core–shell nanocomposite and the synergistic effect between MnO_2_ and Fe_3_O_4_. All these results reveal the MnO_2_/Fe_3_O_4_/diatomite composite is an effective, environmentally friendly and inexpensive Fenton-like catalyst for the removal of organic pollutants.

## Supporting Information

File 1 Additional experimental data.
