# Supplementary material for: Synthesis of a MnO2/Fe3O4/diatomite nanocomposite as an efficient heterogeneous Fenton-like catalyst for methylene blue degradation
Source: Beilstein J Nanotechnol. 2018 Jul 6;9:1940–50. doi: 10.3762/bjnano.9.185 (PMC6036966; doi:10.3762/bjnano.9.185)
Supplement: File 1 — Additional experimental data. [file Beilstein_J_Nanotechnol-09-1940-s001.pdf]

Supporting Information

for

Synthesis of a MnO_2/Fe_3O_4/diatomite nanocomposite as an efficient heterogeneous Fenton-like catalyst for methylene blue degradation

Zishun Li‡[1,2], Xuekun Tang‡[1,2], Kun Liu*[1,2], Jing Huang[1,2], Yueyang Xu[1], Qian Peng[1,2] and Minlin Ao [1,2]

Address: [1]School of Minerals Processing and Bioengineering, Central South University, Changsha 410083, China and [2]Hunan Key Laboratory of Mineral Materials and Application, Central South University, Changsha 410083, China

Email: Kun Liu* - kliu@csu.edu.cn

* Corresponding author

‡ Equal contributors

Additional experimental data

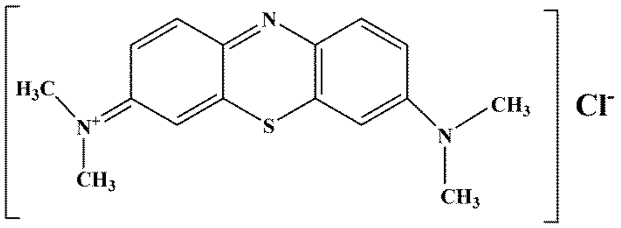

Figure S1: The chemical structure of methylene blue.

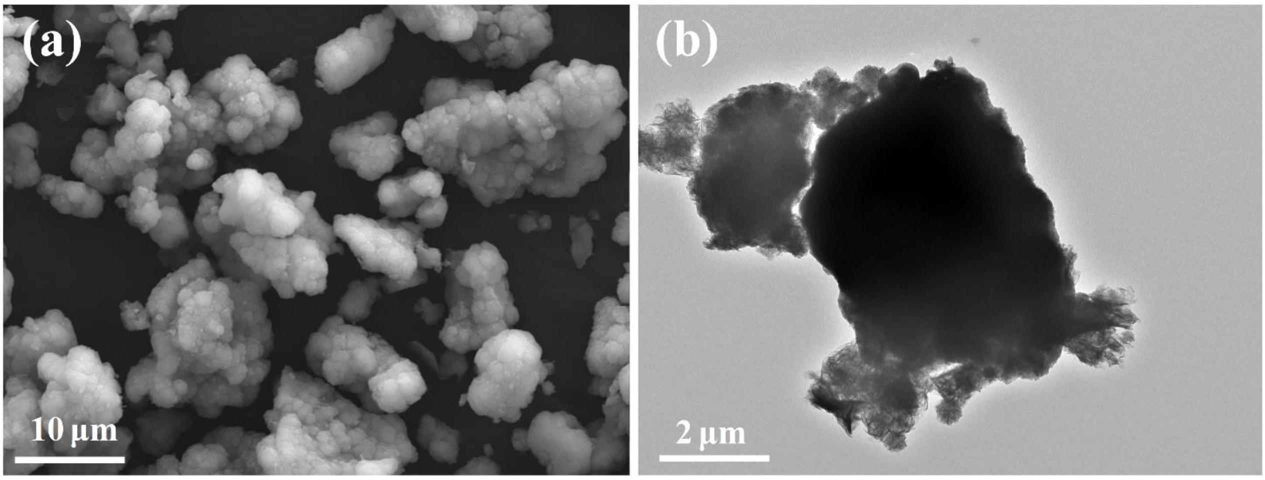

Figure S2: (a) SEM image and (b) TEM image of pure MnO_2 particles.
